# Prevalence and Risk Factors of Latent Tuberculosis Infection (LTBI) in Patients with Type 2 Diabetes Mellitus (T2DM)

**DOI:** 10.3390/ijerph18010305

**Published:** 2021-01-04

**Authors:** Phan Ai Ping, Rosnani Zakaria, Md Asiful Islam, Lili Husniati Yaacob, Rosediani Muhamad, Wan Mohamad Zahiruddin Wan Mohamad, Harmy Mohamed Yusoff

**Affiliations:** 1 Department of Family Medicine, School of Medical Sciences, Universiti Sains Malaysia, Kubang Kerian 16150, Kelantan, Malaysia; aiping1982@yahoo.com (P.A.P.); husniati@usm.my (L.H.Y.); drrosediani@gmail.com (R.M.); 2 Department of Haematology, School of Medical Sciences, Universiti Sains Malaysia, Kubang Kerian 16150, Kelantan, Malaysia; 3 Department of Community Medicine, School of Medical Sciences, Universiti Sains Malaysia, Kubang Kerian 16150, Kelantan, Malaysia; drzahir@usm.my; 4 Faculty of Medicine, Universiti Sultan Zainal Abidin, Kuala Terengganu 21300, Terengganu, Malaysia; harmyusoff@unisza.edu.my

**Keywords:** latent tuberculosis infection, type 2 diabetes mellitus, tuberculin skin test, prevalence, risk factors

## Abstract

Type 2 diabetes mellitus (T2DM) and tuberculosis (TB) together impose a high disease burden in terms of both mortality and health economics worldwide. The objective of this study was to estimate the prevalence and risk factors of latent TB infection (LTBI) in patients with T2DM in Malaysia. A cross-sectional study was performed, and adult T2DM patients (n = 299) were included. Simple and multiple logistic regression analyses were performed to identify the LTBI-associated risk factors in patients with T2DM. Multiple logistic regression was used to estimate adjusted odds ratios (aOR) and 95% confidence intervals (CIs) between T2DM and LTBI and was adjusted for potential confounders. The prevalence of LTBI in patients with T2DM was 11.4% (95% CI: 8.0–15.0%). There was no significant difference in the socio-demographic characteristics between LTBI and non-LTBI subjects. No significant difference in the smoking status, the duration of smoking, and the duration of T2DM, HbA1c, or treatments was observed. Interestingly, a higher level of education was observed to be associated with a lower prevalence of LTBI in T2DM patients (aOR: 0.08, 95% CI: 0.01–0.70, *p* = 0.02). Although the prevalence of LTBI in T2DM was low, it is important to screen for it in T2DM patients due to the risk of developing severe active TB.

## 1. Introduction

In 2018, 10 million people developed active TB [non-human immunodeficiency virus (HIV) TB], and 1.2 million of them died [1]. Latent tuberculosis infection (LTBI) is recognized as a state when a person is infected with *Mycobacterium tuberculosis* without the representation of any clinical manifestations of active TB [2,3]. It was estimated that globally, approximately 1.7 billion people were latently infected with *Mycobacterium tuberculosis* in the year 2014 [4]. The exact mechanism of the development of LTBI is still unclear; however, there is growing evidence suggesting that the risk of reactivation of LTBI into an active TB is greater in some noncommunicable diseases which affect the function of the immune system. Even though the rate of latent TB reactivation is around 10% [5,6], the risk of reactivation is many folds higher in immunosuppressed patients and patients with type 2 diabetes mellitus (T2DM) [7,8,9].

T2DM is still considered as a steadily increasing epidemic global disease burden, with 382 million worldwide sufferers in 2014 and a projected 592 million sufferers by the year 2035 [10,11]. In a meta-analysis, it was observed that T2DM increased the risk of being diagnosed with active TB by three-fold, and 95% of the TB patients were from low- and middle-income countries [12]. Increased incidence of T2DM has also been projected, which may substantially increase the economic impact of this double disease burden [13,14]. Thus, both TB and T2DM are currently amongst the main global public health priorities. Based on the latest (2018) WHO data on Malaysia, over 25,000 people have developed active TB with an incidence rate of 92 per 100,000 and over 2.4 million people are projected to be diabetic by the year 2030 [15,16].

Although researchers have established a relationship between active TB and T2DM, it is yet unclear the extent of the prevalence of T2DM in LTBI and if this comorbid condition increases the risk of developing LTBI. Identifying LTBI (who are at greater risk of reactivation to active TB) in patients with T2DM is an imperative measure as adequate prophylaxis can prevent TB reactivation significantly [6,17]. There are few studies that have explored the relationship between T2DM and LTBI thus far; therefore, the objective of this study was to estimate the prevalence and risk factors of LTBI in Malaysian patients with T2DM.

## 2. Materials and Methods

### 2.1. Study Design and Participants

In this cross-sectional study, adult patients (>18 years) with confirmed T2DM by clinical and blood parameters (fasting blood sugar level and HbA1c) were recruited from Hospital Universiti Sains Malaysia (USM) using the systematic random sampling method in the ratio 1:1 based on the attendance list at the outpatient clinic. Hospital USM is situated between the suburban and rural districts, much like other places in Kelantan state and other residential areas in Peninsular Malaysia. Patients were excluded if they were already diagnosed with TB or had symptoms suggestive of TB, were immunocompromised patients (i.e., HIV-infected), or were on immunosuppressive or on immune-modulatory medications (i.e., >15 mg/day prednisolone for ≥1 month or taking tumor necrosis factor-α antagonists). They were then interviewed using a questionnaire that includes information on gender, ethnicity, marital status, education level, occupation, monthly income, smoking status, age, and smoking duration. On the same day, they underwent the Mantoux test/Tuberculin test, and they were checked for the presence of a Bacillus Calmette–Guérin (BCG) scar. All participants were given an appointment to be seen again after three days. Written informed consents were obtained from the study participants following the ethics approval from the Human Research Ethics Committee, Universiti Sains Malaysia [USMKK/PPP/JEPeM 266.3.(7)].

### 2.2. Sample Size Calculation

The sample size was calculated based on the prevalence of 42% previously reported in a study on LTBI prevalence among diabetic patients in Spain [18]. Using a precision of 6% at the 5% significance level, a total of 325 subjects was required after considering a non-response rate of 20%.

### 2.3. Tuberculin Skin Test (TST) and Interpretation

The standard one-step tuberculin test (TST) was performed, which consisted of an intracutaneous injection of 0.1 mL (5 tuberculin units) of purified protein derivative (Tuberkulin RT 23, Statens Serum Institute, Copenhagen, Denmark) on the volar side of the forearm. Any induration was measured in millimeters after 72 h of the injection. The indurated area referred to the raised region, not the surrounding erythema. The “pen technique” was used for distinguishing the indurated area from the surrounding erythema, where a line was lightly drawn with a pen in the horizontal and vertical planes until the edge of the induration was reached. This procedure was carried out by the well-trained staff nurses using the Mantoux technique. To determine the size of the reaction, the induration was measured transversely to the long axis of the forearm from the most medial point. Generally, an induration of ≥10 mm was considered positive and ≥5 mm was considered positive only in case of recent contact with a TB-positive person. Additionally, the positive subjects were assessed by clinical examinations and a chest X-ray (CXR). Patients with a positive TST without clinical evidence of active TB and with a normal CXR were considered to be LTBI. None of the participants had clinical or radiological evidence of active TB.

### 2.4. Risk Factor Assessment

From the TST result, patients were assigned into two groups: TST-positive (LTBI) or TST-negative (non-LTBI). Data on the duration of T2DM, the HbA1c level (below 6.5%, 6.5–7.0%, and above 7.0%), the diet-controlled only factors, monotherapy with oral hypoglycemic agents (OHA), the use of insulin alone, and the use of a combination of OHA and insulin were verified using hospital records and information was taken during the first interview. The LTBI patients were offered an appointment with a TB specialist, and the non-LTBI patients were given an appointment for a yearly chest X-ray.

### 2.5. Statistical Analysis

All descriptive data were reported as mean and percentages. Continuous data were described as mean, median, and standard deviation (SD). Descriptive statistics were used to determine the prevalence of LTBI in patients with T2DM. Simple and multiple logistic regression analyses were performed to identify the LTBI-associated risk factors in patients with T2DM. Bivariate analyses were performed to determine which independent variables were associated with LTBI. Variables with *p*-value of <0.25 and clinically significant variables were included in the multiple logistic regression analysis. From the multiple logistic regression, we obtained estimate adjusted odds ratios (aOR) and 95% confidence intervals (CIs) between T2DM and LTBI, which were adjusted for potential confounders. A *p*-value of <0.05 was defined as the level of statistical significance. Data analyses were performed using the Statistical Package for Social Sciences (SPSS) version 27 software (IBM Corporation, Armonk, NY, USA).

## 3. Results

Initially, 325 eligible patients with T2DM were recruited; however, only 299 patients were finally included in this study, as these patients came up during the 72-h follow-up for the Mantoux test reading. The prevalence of LTBI in patients with T2DM was 11.4% (95% CI: 8.0–15.0%). Socio-demographic characteristics of the participants are presented in Table 1. In brief, there was no significant difference in the mean ages of patients with LTBI vs. non-LTBI (57.7 ± 9.5 vs. 57.24 ± 9.1). The male female ratio was 1:1. The majority of the participants were married (91.6%) and of Malay ethnicity (86.6%). There was no significant difference on the educational levels, occupation or monthly income between LTBI and non-LTBI subjects. There were no significant differences for duration of T2DM, smoking status, duration of smoking, HbA1c result, previous BCG vaccination, and treatments between the patients with LTBI and non-LTBI (Table 2). A significant association was observed from the multiple logistic regression between the education level and LTBI. Higher education levels (degree/master/PhD) were associated with a lower prevalence of LTBI (Table 3).

## 4. Discussion

This cross-sectional study estimated the prevalence of LTBI as being 11.4% in patients with T2DM. T2DM is considered as a well-established risk factor for active TB. Although the association between T2DM and LTBI has been hypothesized previously, there is a limitation of evidence from epidemiologic studies. In a cross-sectional study from the USA, a similar prevalence of LTBI [11.6% (95% CI: 7.9–15.3)] was observed, although the type of diabetes was not addressed in the study [19]. In comparison to our Malaysian cohort, the prevalence of LTBI was high in some other T2DM populations, including Egyptian (21.6%) [20] and Singaporean (28.2%, 95.5% T2DM patients) populations [21]. An extremely high prevalence of LTBI was observed in a Mexican T2DM cohort (51.3%) [22] and in a group of Chinese residents of old age homes (mean age 82 years) (52.5%) with diabetes [23].

Interestingly, our study determined that a lower prevalence of LTBI in patients with T2DM was associated with higher levels of education, although this result should be considered with caution as it is based on a small sample size. From our observation, subjects with lower levels of education have higher chances of exposure to TB by working in construction sites or factory settings where they had increased risk to the exposure of migrant workers, especially from high TB burden countries. In contrast, patients with high levels of education mostly work in offices and in other managerial working environments where their exposure to crowded places and poor sanitation areas was less high. They also have more of an ability and knowledge for monitoring their T2DM and had more frequent medical screenings.

There are a few established molecular bases of the prevalence of LTBI in T2DM. In patients with T2DM, the immune system is compromised, which actually favors the reactivation of latent TB to an active status [19,24]. This possibly happens through the activities of adipocytokines—cytokines produced by the adipose tissues of T2DM patients. Adipokines induce inflammatory processes in patients with T2DM and dysregulation of this cytokine has an impact on increased risk of developing LTBI in patients with T2DM [25].

There are some notable strengths in this study. To the best of our knowledge, this is the first cross-sectional study aiming to estimate the prevalence of LTBI in patients with T2DM in a Malaysian population. Previously, another study conducted by Swarna Nantha et al. [26] included both diabetic and non-diabetic patients in a case-control setting and the OR of LTBI in diabetic patients was estimated to be 1.88 (95% CI: 1.22–2.82) compared to non-diabetic patients. The diagnosis of T2DM was diagnostically confirmed and was not on a self-reported basis. The sample size was based on the sample size calculation; therefore, the outcomes of this study is robust. Although most of our participants were vaccinated with BCG and this can cross-react with the TST test with false-positive results, the participants had their BCG vaccination more than 15 years prior to our study, which reduced the chance of false positive results [27]. In our study, there are some limitations to address. One of the main limitations is that the participants were screened through TST only, which is a one-step TST test and therefore has less specificity, especially in immunocompromised patients. However, patients that agreed to the prophylactic treatment with rifampicin for six months underwent an interferon-γ release assay (IGRA) test for re-confirmation of their LTBI status. Unfortunately, less than 20% of patients agreed to prophylactic treatment, as most patients were reluctant to start the treatment because of its long duration time. All of the LTBI positive by TST were found to be positive by IGRA as well; however, as the number of overlapping positive results was very small, this has to be interpreted with caution. Some of the socio-demographic information collected such as smoking status or any previous TB contact may be undermined by the limitations of stigmatization. Due to the nature of the cross-sectional study design, it is difficult to draw inferences of causality with certainty.

The confirmation of LTBI in patients with T2DM is notoriously challenging [28]. The available methods—(i) TST and (ii) IGRAs—are based on patients’ immunological response to a stimulus, where the immunological response in patients with T2DM may be impaired which ultimately affects the results, unfortunately. TST has a poor specificity and boosting phenomenon as well whereby the initial response towards TST can be negative, whereas IGRA show higher rates of false positive results [29,30]. Therefore, improvements in the diagnosis of LTBI in patients with T2DM are indeed warranted.

## 5. Conclusions

Although the prevalence of LTBI in T2DM was low, it is important to screen for it in patients with T2DM due to the risk of developing severe active TB. Prospective studies are warranted to further investigate the temporal relationship between T2DM and both LTBI and disease onset. In the future, observational studies with long-term follow-up would be interesting to carry out to investigate the reactivation of LTBI in patients with T2DM.

## Figures and Tables

**Table 1:** Socio-demographic characteristics of patients with latent tuberculosis infection (LTBI) and non-LTBI.

Characteristics	Non-LTBI (n = 265)		LTBI (n = 34)		*p*-Value
	n (%)	Mean ± SD	n (%)	Mean ± SD	
**Age**		57.7 ± 9.5		57.2 ± 9.1	0.75 ^a^
**Gender**					
Male	125 (47.2)		18 (53.0)		0.59 ^c^
Female	140 (52.8)		16 (47.0)		
**Ethnicity**					
Malay	227 (85.7)		32 (94.0)		0.11 ^b^
Chinese	35 (13.2)		1 (3.0)		
Indian	1 (0.3)		1 (3.0)		
Others	2 (0.8)		0 (0.0)		
**Marital status**					
Married	242 (91.3)		32 (94.2)		0.40 ^b^
Single	11 (4.1)		0 (0.0)		
Divorced	2 (0.8)		1 (2.9)		
Widow	10 (3.8)		1 (2.9)		
**Educational level**					
None	47 (17.7)		10 (29.4)		0.19 ^b^
Primary	53 (20.0)		5 (14.7)		
Secondary	99 (37.4)		15 (44.1)		
Diploma	27 (10.2)		3 (8.8)		
Degree/Master	39 (14.7)		1 (3.0)		
**Occupation**					
Unemployed	107 (40.4)		17 (50.0)		0.47 ^b^
Self-employed	71 (26.8)		7 (20.6)		
Labour work	46 (17.4)		6 (17.6)		
Managerial	17 (6.4)		0 (0.0)		
Pensioner	24 (9.0)		4 (11.8)		
**Monthly income**		1508.9 ± 1975.4		1599.6 ± 3705.6	0.82 ^a^

^a^ Numerical variable using Independent *t*-test, ^b^ Categorical variable using Chi-square test, ^c^ Fisher’s exact test.

**Table 2:** Risk factors between patients with LTBI and non-LTBI.

Risk Factors	Non-LTBI		LTBI		*p*-Value
	n (%)	Mean ± SD	n (%)	Mean ± SD	
**Smoking status**					
Non-smoker	225 (85.0)		29 (85.3)		>0.95 ^b^
Smoker	19 (7.1)		2 (5.9)		
Ex-smoker	21 (7.9)		3 (8.8)		
**Duration of smoking (years)**		21.2 ± 10.0		19.5 ± 0.7	0.68 ^a^
**Duration of T2DM (months)**		108.7 ± 83.3		104.2 ± 82.3	0.77 ^a^
**HbA1c (%)**		8.5 ± 2.2		8.8 ± 2.0	0.49 ^a^
**BCG scar**					
No	2 (0.8)		0 (0.0)		>0.95 ^c^
Yes	263 (99.2)		34 (100.0)		
**Treatments**					
Diet alone	3 (1.1)		0 (0.0)		0.45 ^b^
OHA	164 (61.9)		18 (53.0)		
Insulin	98 (37.0)		16 (47.0)		

^a^ Numerical variable using Independent *t*-test, ^b^ Categorical variable using Chi-square test, ^c^ Fisher’s exact test, T2DM: type 2 diabetes mellitus, OHA: oral hypoglycemic agent.

**Table 3:** Associated factors for LTBI by multiple logistic regression.

Factors	Regression Coefficient	Adjusted Odds Ratio (95% CI)	*p*-Value
**Age**	−0.11	0.99 (0.94, 1.04)	0.66
**Gender**			
Male		1.0	
Female	−0.398	0.67 (0.29, 1.56)	0.36
**Ethnicity**			
Malay		1.0	
Chinese	−1.675	0.19 (0.02, 1.44)	0.11
Indian	1.904	6.71 (0.39, 114.72)	0.19
Others	−19.724	0.000	>0.95
**Educational level**			
None		1.0	
Primary	−1.186	0.30 (0.09, 1.06)	0.06
Secondary	−0.753	0.47 (0.19, 1.20)	0.11
Diploma	−0.936	0.39 (0.10, 1.58)	0.19
Degree/Master/PhD	−2.459	0.09 (0.01, 0.71)	0.02
**Smoking status**			
Non-smoker		1.0	
Smoker	−0.077	0.93 (0.17, 4.98)	0.93
Ex-smoker	−0.556	0.57 (0.10, 3.35)	0.54
**Duration of T2DM**	0.000	1.00 (0.99, 1.01)	0.91
**HbA1c**	−0.021	0.98 (0.80, 1.20)	0.84
**Treatments**			
Diet alone		1.0	
OHA/Insulin alone and/or combination	0.641	1.90 (0.86, 4.18)	0.11

## Data Availability

The data presented in this study are available in article.
